# Inactivation of FOXA2 by Respiratory Bacterial Pathogens and Dysregulation of Pulmonary Mucus Homeostasis

**DOI:** 10.3389/fimmu.2020.00515

**Published:** 2020-03-25

**Authors:** Woosuk Choi, Shawn Choe, Gee W. Lau

**Affiliations:** Department of Pathobiology, University of Illinois at Urbana-Champaign, Urbana, IL, United States

**Keywords:** chronic lung diseases, mucus homeostasis, mucociliary clearance, FOXA2, EGFR, STAT6, MUC5AC, MUC5B

## Abstract

Forkhead box (FOX) proteins are transcriptional factors that regulate various cellular processes. This minireview provides an overview of FOXA2 functions, with a special emphasis on the regulation airway mucus homeostasis in both healthy and diseased lungs. FOXA2 plays crucial roles during lung morphogenesis, surfactant protein production, goblet cell differentiation and mucin expression. In healthy airways, FOXA2 exerts a tight control over goblet cell development and mucin biosynthesis. However, in diseased airways, microbial infections and proinflammatory responses deplete FOXA2 expression, resulting in uncontrolled goblet cell hyperplasia and metaplasia, mucus hypersecretion, and impaired mucociliary clearance of pathogens. Furthermore, accumulated mucus clogs the airways and creates a niche environment for persistent microbial colonization and infection, leading to acute exacerbation and deterioration of pulmonary function in patients with chronic lung diseases. Various studies have shown that FOXA2 inhibition is mediated through induction of antagonistic EGFR and IL-13R-STAT6 signaling pathways as well as through posttranslational modifications induced by microbial infections. An improved understanding of how bacterial pathogens inactivate FOXA2 may pave the way for developing therapeutics that preserve the protein's function, which in turn, will improve the mucus status and mucociliary clearance of pathogens, reduce microbial-mediated acute exacerbation and restore lung function in patients with chronic lung diseases.

## Introduction

The *Forkhead* (*fox)* genes encode evolutionarily-conserved transcriptional regulators characterized by a winged-helix DNA-binding domain (DBD), called the forkhead box. Members of the FOX family have divergent roles, including embryonic development, cell survival, proliferation, differentiation, and energy homeostasis (1). Initially discovered in *Drosophila melanogaster*, a mutation in the *fox* gene generated a homeotic transformation of foregut into head that originates the nomenclature “forked head” (2). Over 100 FOX members have been identified, with 50 human *fox* genes cataloged into 19 subgroups from FOXA to FOXS (3). The FOXA subclass was the first discovered in mammals (4).

## Structure and Function Of Foxa2

FOXA1, FOXA2, and FOXA3 were originally identified in rat liver, the so-called hepatocyte nuclear factor 3 (HNF3)-α, -β, and -γ, respectively (4). The conserved domains of FOXA2 were originally analyzed (2), and the DBD was found to be the most evolutionarily conserved among the FOXA members, with an unique AKT/PKB phosphorylation site at the threonine (Thr)156 (5). The DBD of FOXA is structurally similar to H1/H5 linker histones capable of unwinding chromatin, which enables the recruitment of other transcriptional cofactors to the promoter (6, 7). The C-terminus of FOXA interacts with histones H3/H4 within nucleosome to support the opening of chromatin by the DBD (8). FOXA members regulate the expression of target genes by displacing histones from chromatin and serving as transcription factors at the enhancer region of the promotor (9, 10).

## Modulation Of Foxa2 Activity by Post-Translational Modifications

**Figure 1:**
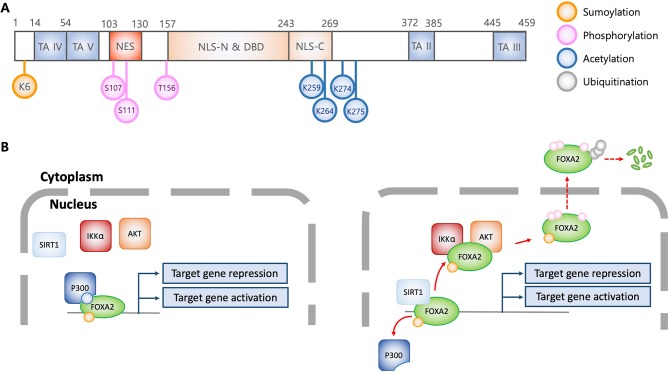
Structural and functional characteristics of FOXA2 in association with posttranslational modification. **(A)** Schematic diagram of FOXA2 (adapted from J Biol Chem 2009, 284:24816-24; and Mol Cell Biol 1992, 12:3723-3732). Diagram shows functional domains of FOXA2. Colorized circles indicate post-translationally modified amino acid residues which alter FOXA2 functions. TA, transactivation domain; NES, nuclear export signal; NLS, Nuclear localization signal; DBD, DNA binding domain; K, lysine; S, serine; T, threonine. **(B)** Regulation of transcriptional activity and stability of FOXA2 by posttranslational modifications. Acetylation by P300 allows FOXA2 to be functionally active. In contrast, SIRT1 deacetylates FOXA2, leading to nuclear export via AKT and IKKα-mediated phosphorylation, and subsequently, ubiquitination and degradation. Sumoylation enhances the stability of nuclear FOXA2, which is diminished by ubiquitination, resulting in FOXA2 degradation.

FOXA transcription factors are abundantly expressed in liver and regulate metabolic homeostasis (4). During hypoglycemia, low plasma insulin activates FOXA2 to elevate the transcription of genes encoding metabolic enzymes involved in fatty acid oxidation and ketogenesis, supplying energy for gluconeogenesis and maintaining brain function, respectively. However, excess blood glucose elevates plasma insulin, which inactivates FOXA2 and decreases the expression of enzymes involved in gluconeogenesis (11, 12). This is supported by studies that show inactivation of hepatic FOXA2 in hyperinsulinemic *ob/ob* and *db/db* mice and in diet-induced obese mice (13). Insulin promotes nuclear export of FOXA2 through AKT-mediated phosphorylation at Thr156 (Figures 1A,B) (5). Cells expressing the phosphorylation-deficient FOXA2-T156A are unresponsive to insulin-induced AKT, resulting in constitutive nuclear localization. Interestingly, DNA-binding ability of FOXA2 is not altered by insulin and phosphorylation-deficiency, suggesting that phosphorylation at Thr156 does not regulate transcriptional activity. Further study indicates that in the presence of insulin, phosphorylation at Thr156 inactivates transcriptional function and induces nuclear exclusion of FOXA2. Nuclear export of FOXA2 is dependent upon nuclear export factor CRM1, which recognizes the leucin-rich NES consensus sequence L*X*_2,3_(L/I/V/F/M)*X*_2,3_L*X*(L/I) within amino acids 106-111 (LSPSLS) of FOXA2 (14). In contrast, others have reported that FOXA2 is constitutively localized to the nucleus, independent of the metabolic conditions (15, 16). The aforementioned discrepancies might be caused by distinct experimental conditions, including differences in transgenic obese mouse strains, feeding conditions, and immortalized hepatic cell lines. Subsequently, it was revealed that FOXA2 function could be modulated by IKKα-mediated phosphorylation on Ser107/111 (Figure 1A) (17). Both Thr156 and Ser107/111 residues are located within the nuclear localization signal domain and the nuclear export signal domain, respectively (Figure 1A), implying that phosphorylation of these residues dictates subcellular localization of FOXA2 (14).

Acetylation and deacetylation also compete to modulate the transcriptional activity of FOXA2. Insulin induces the SIRT1 deacetylase to deacetylate Lys259 on FOXA2 (Figure 1A), which attenuates target gene expression and increases export from the nuclei in hepatocytes. In contrast, glucagon induces FOXA2 acetylation through P300 acetyltransferase, which leads to nuclear accumulation and increased expression of target genes (12). The acetylation-deficient FOXA2-K259R has attenuated DNA binding ability, and is sequestered to the cytoplasm for degradation. As discussed above, FOXA2-T156A is sequestered in the nucleus; however, hepatic cells transfected with *FOXA2-T156A-K259R* exhibit similar phenotype to those transfected with *FOXA2-K259R*, suggesting that acetylation/deacetylation diminish phosphorylation event, and therefore, confer a dominant phenotype (12). Interestingly, it has been reported that during nutrient-deprivation, deacetylation by SIRT1 only attenuates FOXA2's transcriptional activity but not its nuclear export for degradation by the proteasome (11).

Sumoylation and ubiquitination also compete for maintenance and degradation of FOXA2 (18) Sumoylation of the Lys6 by PIAS stabilizes FOXA2's transcriptional activity. In contrast, polyubiquitination facilitates FOXA2 degradation by proteasome. Sumoylation-resistance FOXA2-K6R is more susceptible to ubiquitination and rapid degradation, suggesting that additional lysine residues might be ubiquitinated. Notably, although sumoylation increases both stability and transcriptional activity of FOXA2, nuclear localization is not affected.

## Role of Foxa2 in Lung Development and Homeostasis

FOXA2 is required for lung development. During mouse embryogenesis, FOXA2 is initially expressed in the primitive streak and in the node on embryonic day 6.5 (E6.5), inducing gastrulation. By E7.5, FOXA2 is highly expressed in both mesoderm and endoderm, and thereafter, persistently expressed throughout development and in adult endoderm-derived tissues, including the lung (19). After E10-E11, lung morphogenesis is facilitated by the spatiotemporal expression of FOXA2 restricted to respiratory epithelium. By E12.5, mouse embryo develops conducting airways and alveolar epithelial cells, and forms lung buds during late embryonic development (20). In contrast, *FOXA2* null mouse embryo shows lethality on E11-E12, with severe defects in all three germ layers before the initiation of lung morphogenesis (21). The conditional loss of FOXA2 on E12.5 disrupts branching formation and airway epithelial cell differentiation, resulting in the dilation of distal airways. In addition, postnatal (PN) lungs of *foxa1* null allele and lung epithelium-specific *foxa2* depleted mice (*foxa1*^−/−^*/foxa2*^Δ/Δ^*)* exhibit regressed formation of alveolar and peripheral lung saccules by PN day 3 (PN3), and extensive airspace enlargement with mucin glycoprotein overexpression by PN10–PN20 (20).

In postnatal lungs, FOXA2 is constitutively expressed in subsets of respiratory epithelial cells and transcriptional controlled genes encoding club cell specific protein CC10 (22), surfactant proteins (SP) (23), thyroid transcription factor-1 (TTF-1) (24), and mucins MUC5AC and MUC5B (25, 26). Pulmonary surfactant is composed of 90% phospholipids and 10% proteins, including SP-A, SP-B, SP-C, and SP-D secreted by the type II alveolar cells and non-ciliated terminal bronchiolar Club cells. Together with phospholipids, SP-B and SP-C provide critical surface tension-lowering properties that reduce the work of breathing and maintain airspace patency. FOXA2^Δ/Δ^ mice show significantly reduced SP-B expression with deteriorating respiratory distress syndrome (27). In contrast to upregulation of CC10, SFTPB, and TTF-1, FOXA2 represses the transcription of mucin genes. Conditional deletion of FOXA2 in the mouse respiratory epithelium causes airspace enlargement, goblet cell hyperplasia, increased mucin expression and neutrophil infiltration (25). Collectively, the aforementioned studies indicate crucial roles of FOXA2 in regulating embryonic lung development and postnatal lung homeostasis.

## Foxa2 and Mucus Homeostasis

**Figure 2:**
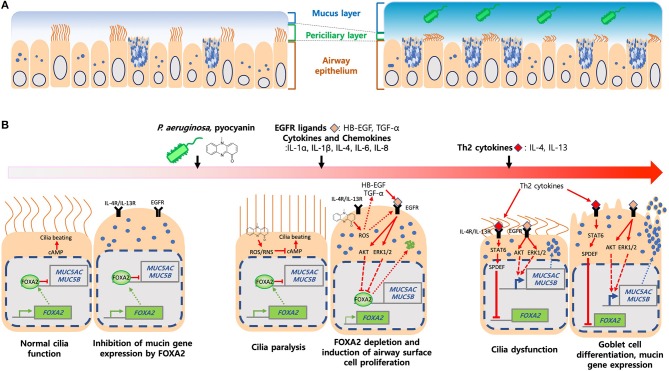
Induction of mucus hypersecretion by *P. aeruginosa* pyocyanin. **(A)** Composition of mucus layers in heathy and diseased airways. **(B)** *P. aeruginosa* and its virulence factors, especially pyocyanin, stimulate excessive ROS, chemokines and cytokines and, ligands that activate IL-4R/IL-13R-STAT6-SPDEF, and EGFR-AKT/ERK1/2-mediated signaling pathways. Both kinase cascades converged to inhibit FOXA2, resulting in over proliferation and differentiation of airway epithelial (ciliated and club) cells to mucus-secreting goblet cells. Excessive mucus and failure in the clearance exacerbates airway obstruction and microbial colonization and infection.

The apical surface of healthy airways is covered by the airway surface liquid (ASL) composed of mucin glycoproteins, antimicrobial peptides and proteins, innate immune cells, signaling molecules, and enzymes (28). ASL is bilayer, with the periciliary layer sandwiched between the top mucus gel layer and the bottom airway epithelium, forming the “gel-on-brush” structure (29) (Figure 2A). The periciliary layer is filled with hydrogel that provides space for ciliary beating and supports mucociliary clearance. Within the mobile mucus layer, MUC5AC and MUC5B are the predominant mucins that provide viscosity and gel-forming properties to mucus, trapping inhaled pathogens and irritants. Mucins also keep moisture in the airway epithelium (30), which help to maintain the periciliary layer and mucociliary clearance. MUC5AC is produced by the goblet cells while MUC5B is expressed in submucosal glands of trachea and bronchi, and in surface secretory cells throughout the airway down to the level of preterminal bronchioles (31, 32). MUC5AC-overexpressing mice are more resistant to PR8/H1N1 influenza virus (33). Similarly, MUC5B-deficient mice are more susceptible to lung infection with increased mortality caused by ensuing bacteremia (34).

FOXA2 deficiency causes pulmonary eosinophilia, recruitment of inflammatory immune cells and upregulation of IL-4, IL-13, IL-33, CCL-17, and CCL-20 that promote Th2 cell differentiation, goblet cell hyperplasia and metaplasia, and mucus hypersecretion (25, 35). These suggest that FOXA2 regulates airway mucus homeostasis by counteracting the effects of IL-4 and IL-13 (36, 37). Metaplastic effects of IL-4 and IL-13 are mediated through the STAT6 (35) and its downstream effector SPDEF (25, 38, 39). MUC5Ac and MUC5B expression is dependent on SPDEF (38, 40). IL-13-stimulated airway epithelial cells show decreased *FOXA2* transcript, a process mediated by SPDEF (41). Conditional induction of SPDEF within transgenic mouse airways downregulates the *foxa2* gene, resulting in goblet cell hyperplasia (39). These findings indicate that FOXA2 and SPDEF compete to regulate the expression of MUC5AC and MUC5B.

Th2 cytokines also amplify the mucus-inducing EGFR signaling, which is highly activated in cystic fibrosis (CF), chronic obstructive pulmonary disease (COPD) and asthma (42, 43). In response to IL-4, IL-5, and IL-13, bronchial epithelial and immune cells produce ligands (EGF, TGF-α, amphiregulin) that induce EGFR in an autocrine manner (41, 44–46), which subsequently, activates the downstream cRAF-MEK-ERK and PI3K-AKT signaling cascades that inhibit FOXA2 and increase MUC5AC and MUC5B production (47). Interestingly, these pathways have distinct effects, with PI3K-AKT augments cell proliferation while cRAF-MEK-ERK directly enhances goblet cell metaplasia and mucin synthesis (48).

Notch signaling regulates cell-cell communication and differentiation of airway basal cells into secretory and ciliated cells (49). Both Notch1 and Notch2 are required for goblet cell development (50, 51). Interestingly, Notch ligands promotes goblet cell metaplasia independent of the IL-13R-STAT6 axis. It is unknown if Notch induction depletes FOXA2 expression during goblet cell development in diseased airways.

Interestingly, *MUC5B* gene transcription is differentially-regulated by FOXA2. Inhibition of FOXA2 by bacterial pathogens elevates MUC5B expression (26, 52–54). In contrast, FOXA2 positively regulates MUC5B expression in both idiopathic pulmonary fibrosis (55) and asthma (56), most likely caused by polymorphism in the *MUC5B* promoter (57). Additionally, inhibition of FOXA2 by IL-13 results in different inhibitory kinetics on *MUC5B* expression in the air-liquid interface (ALI) culture of airway cells vs. in mouse lungs (41), suggesting that additional mediators may modulate mucin production in an intact lung. Collectively, these findings suggest that regulatory activity of FOXA2 on *MUC5B* may vary, depending on disease context and additional interacting factors.

## Foxa2 Inactivation by Respiratory Bacterial Pathogens

As previously discussed, excessive mucus causes airway obstruction, narrowing and airflow limitation in chronic lung diseases. Significantly, FOXA2 expression is depleted in airways of patients with bronchopulmonary dysplasia, bronchiectasis (25), and asthma (58). Cigarette smoking, the most important etiologic agent in COPD, directly suppress FOXA2 expression (59). Accumulated mucus allows microbes to thrive, resulting in persistent inflammation, acute exacerbation (60), and lung function impairment (61), with increased morbidity, and mortality (62). Among the bacterial pathogens, *Staphylococcus aureus* and *Pseudomonas aeruginosa (PA)* are the most important in young CF patients, but in adulthood, *PA* predominates (63). *Chlamydophila pneumoniae* and *Mycoplasma pneumoniae* are the most important in asthma induction and acute exacerbation (64). *Streptococcus pneumoniae, Haemophilus influenza*, and *Moraxella catarrhalis* are the most common in COPD. However, in advanced stages of COPD, *PA, M. pneumoniae, H. parainfluenzae*, and *Klebsiella pneumoniae* predominate. Acquisition of *PA* increases episodes of acute exacerbation, especially in COPD patients who received antibiotics and those who require mechanical ventilation. Significantly, a subset of these patients becomes chronically-infected with *PA* (65).

For the remainder of this review, we will focus on the FOXA2 inactivation by *PA* (Figures 2A,B). *PA* forms biofilms in the mucus-rich environments and becomes more resistant to antibiotics and phagocytic clearance. *PA* virulence factors, including pyocyanin (26, 52, 66, 67), LPS (68), flagellin (69, 70), alginate (71) and protease (72) induce mucus overproduction.

Among these aforementioned virulence factors, the tricyclic phenazine pyocyanin, is the most robust mucus inducer (73). Pyocyanin is zwittwerionic, which ionizes at physiological pH, penetrates cell membrane, and increases both intracellular reactive oxygen species (ROS) and nitrogen species (RNS) (74–76). Redox cycling of pyocyanin with intracellular electron donors and acceptors generates ROS/RNS (76, 77). Persistent oxidative stress causes dysfunction of ion pumps, antioxidant proteins and cellular reducing agents, resulting in cytotoxicity (78, 79).

Pyocyanin is important for lung infections (80) and recoverable at 0.1 mM concentrations from both COPD and CF sputa (81). Additionally, the levels of pyocyanin within sputa negatively-correlates with the function of CF lungs (82). Pyocyanin interferes with ciliary beating and mucus transport (81), induces bronchoconstriction (83), and decreases mucus velocity (84, 85). Mouse lungs chronically-exposed to pyocyanin develop goblet cell hyperplasia and metaplasia, peribronchial fibrosis, and alveolar airspace destruction, accompanied by polarization from initially a Th1 response toward a Th2 response dominated by IL-4 and IL-13 secreted by activated macrophages and CD4^+^ T cells, with concomitant influx of neutrophils (52, 66). Many of these pathological features resemble the airways of FOXA2-deficient mice (25). Further studies in the both primary and immortalized human airway cells and in mice demonstrate that pyocyanin depletes FOXA2 expression by activating antagonistic EGFR-PI3K-AKT, EGFR-MEK-ERK and IL-13R-STAT6-SPDEF pathways, resulting in goblet cell hyperplasia and metaplasia and excessive mucins (52, 66). Additionally, pyocyanin activates EGFR directly through ROS or indirectly by inducing the release of proinflammatory cytokines and EGFR ligands from airway cells (86). ROS/RNS generated by pyocyanin post-translationally modify FOXA2 and reduce its binding affinity to the *MUC5B* promoter. Glutathione restores the expression of FOXA2, which inhibits the transcription of *MUC5AC* and *MUC5B* (26). Collectively, these results suggest that pyocyanin inactivates FOXA2 through EGFR-PI3K-AKT, EGFR-MEK-ERK, and IL-13R-STAT6-SPDEF signaling, and post-translational modification of FOXA2.

As for the remaining *PA* virulence factors, LPS activates the Src-dependent Ras-p38MAPK-pp90rsk pathway, leading to mucin overproduction (68). Flagellum binds asialoGM1 and induces a signaling cascade, leading to cleavage of PIP2 by PLC, formation of IP3, Ca^2+^ mobilization, phosphorylation of ERK1/2, and finally, transcription of the *MUC2* gene (70). Flagellum also activates mucin biosynthesis through the NF-κB induced by TLR5-IL-8 signaling (69). The mechanisms underlying mucin induction by exoproteases (72), alginate (71), and their association to FOXA2 inactivation remain uncharacterized.

FOXA2 appears to be an evolutionally-conserved target of inactivation. Besides *PA*, we have shown that *M. pneumoniae* inactivates FOXA2 by inducing both STAT3-STAT6 and EGFR signaling, resulting in overexpression of airway mucins (53). Additional evidences are found in canine species. Because of genetic predisposition and exposure to environmental pollutants and infectious agents, older dogs, especially of smaller breeds, develop lung diseases similar to those in humans. Our recent study in dogs with COPD and chronic bronchitis indicate that infection by *PA* and *Bordetella bronchiseptica*, and by viral-bacterial combination activate the antagonistic STAT6 and EGFR signaling to inhibit FOXA2, resulting in goblet cell hyperplasia and metaplasia and mucus hypersecretion (16).

## Summary

Although many aspects of mucus biology have been explored as therapeutic targets, few drugs are available because of inefficacy and adverse effects (87). Despite its importance, FOXA2 has not been targeted for the development of novel mucoregulators, perhaps due to the complexity in gene regulation, post-translational modifications, and toxicity associated with its regulation of multiple cellular processes. Recently, the peptide ADEL (Ala-Asp-Glu-Leu) was found to relieve bacterial-mediated inflammation and improve lung function while boosting FOXA2 expression (88). Additionally, GLP-1 analogs were shown to reduce mortality and improve lung function in mice with acute obstructive lung disease (89). Our latest results indicate that the GLP-1 analog Exenatide restores FOXA2-regulated airway mucus homeostasis through the GLP1R-PKA-PPARγ-phosphatase signaling, by dephosphorylating key kinases within both STAT6 and EGFR cascades (90). Some factors need to be considered before repurposing the GLP-1 analogs. Systemically-administered GLP-1 analogs could suppress appetite (91). Because patients with muco-obstructive diseases (e.g., CF, COPD) commonly experience inappetence, and in the case of CF patients, nutrient malabsorption (92, 93), GLP-1 analogs may adversely impact the health status of these patients. To minimize systemic toxicity, direct aerosolization should be considered. Also, co-prescribing steroids and appetite stimulants with GLP-1 analogs may boost positive outcome. Finally, detailed mechanistic characterization of how these drugs restore FOXA2 function may lead to new methods of controlling excessive mucus, lowering bacterial burden and improving the quality of life in patients with chronic lung diseases.

## Author Contributions

WC, SC, and GL co-wrote the manuscript.

### Conflict of Interest

The authors declare that the research was conducted in the absence of any commercial or financial relationships that could be construed as a potential conflict of interest.
